# The Role of Pretreatment Serum Neutrophil-to-Lymphocyte Ratio in Hypopharyngeal Cancer Treated with Definitive Chemoradiotherapy: A Pilot Study

**DOI:** 10.1038/s41598-018-38282-z

**Published:** 2019-02-07

**Authors:** Chin Kuo, Wei-Ting Hsueh, Yuan-Hua Wu, Ming-Wei Yang, Yung-Jen Cheng, Tzu-Hui Pao, Mu-Hung Tsai

**Affiliations:** Department of Radiation Oncology, National Cheng Kung University Hospital, College of Medicine, National Cheng Kung University, Tainan, Taiwan

## Abstract

Serum neutrophil-to-lymphocytes ratio (NLR) is a potential predictive and prognostic marker in head and neck cancers. This study aimed to determine the role of pretreatment serum NLR in patients with hypopharyngeal cancer (HPC) treated with definitive chemoradiotherapy. We retrospectively investigated the correlation between clinicopathological parameters and NLR status and analysed its impact on therapeutic response and survival. A total of 120 patients treated at a single institution between 2009 and 2015 were included. The median follow-up time was 24.1 months. High NLR (NLR ≥ 4) was associated with advanced T classification (*p* = 0.01*) and advanced stage (*p* = 0.02*) based on chi-square test. We also found that high pretreatment NLR was correlated with poor treatment response (HR = 2.42, 95% CI: 1.08–5.44, *p* = 0.03*). Pretreatment NLR was also an independent prognostic factor for progression-free survival (HR = 1.71, 95% CI: 1.01–2.90, *p* = 0.046*) and overall survival (HR = 1.99, 95% CI: 1.21–3.28, *p* = 0.01*) while correcting for known prognostic factors. Overall, these findings support that NLR is a potential biomarker for host response to tumour aggressiveness, therapeutic response to chemoradiotherapy and survival in HPC patients. This study is limited by its retrospective nature and further validation is warranted.

## Introduction

Hypopharyngeal carcinoma (HPC) has a poor prognosis. Despite aggressive treatment, three-year overall survival (OS) ranges from 46.9% to 78.8% and three-year progression-free survival (PFS) ranges from 42.0% to 58.4%^1–4^. The gold standard treatment for HPC is total laryngopharyngectomy, which results in unavoidable voice loss and decreased quality of life. As an alternative treatment, the Veterans’ Administration Laryngeal Cancer Study Group trial assessed an organ-preservation approach using definitive chemoradiotherapy, resulting in long-term control without sacrificing the larynx, which had a survival rate similar to that of surgery^5–7^. However, patients with a poor response to definitive chemoradiotherapy may compromise survival with this approach, making major surgery more suitable for these patients. Therefore, pretreatment biomarkers that can help predict response and prognosis are of high clinical value.

Mounting evidence suggests that the tumour microenvironment plays a crucial role in tumour proliferation, invasion, metastases and resistance to treatment^8,9^. It has recently become clear that cancer-related inflammation, including local and systemic inflammation, correlates with therapeutic response and survival in various solid organ malignancies^10,11^. Numerous inflammation-related parameters have been studied, including plasma C-reactive protein, albumin level, Glasgow Prognostic Score, neutrophil-to-lymphocyte ratio (NLR) and platelet-to-lymphocyte ratio (PLR)^10,12,13^. NLR is one the of most easily calculated, inexpensive and reproducible markers of systemic inflammation; its value as a prognostic factor in lung cancer, renal cell carcinoma, gastric cancer and colon cancer has been confirmed in previous studies^14,15^. Previously, two retrospective studies reported high pretreatment NLR correlated with a higher incidence of wound complications, inferior overall survival and inferior disease-free survival in HPC patients receiving surgery^16,17^. However, the value of NLR as a prognostic factor in HPC receiving definitive chemoradiotherapy remains unclear. Thus, further research is needed to elucidate the role of NLR in HPC patients undergoing definitive chemoradiotherapy.

The purpose of this study was to examine the association between pretreatment NLR and clinicopathological parameters and to determine the prognostic significance of pretreatment NLR in HPC patients treated with definitive chemoradiotherapy.

## Results

### Patient characteristics

**Figure 1:**
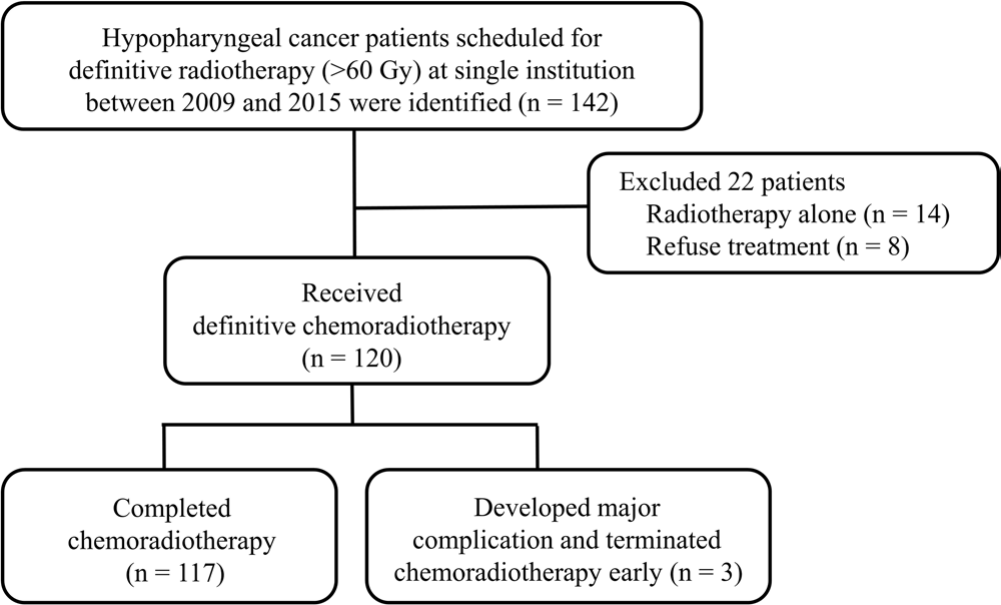
Flowchart of the retrospective study design.

The outcomes of patients with non-metastatic hypopharyngeal cancer undergoing curative definitive radiotherapy (>60 Gy) at a single institution were retrospectively reviewed. A total of 142 patients were reviewed. Of these, 14 were excluded due to treatment using radiotherapy alone, which was considered inadequate. Another 8 patients were excluded due personal factors related to poor compliance. Thus, a total of 120 patients were included in this study (Fig. 1).

The median age at diagnosis was 55 years. The majority of patients were male (n = 117, 97.5%), had a good performance (ECOG performance status 0–1, n = 105, 87.5%) and had none or mild comorbidities (n = 85, 70.8%). Sixteen (13.3%) patients were underweight (BMI < 18.5) and 67 patients (77.9%) were heavy smokers. Most primary tumours were located at the pyriform sinus (n = 97, 80.8%), while 6 (5.0%) patients had a tumour located in the post-cricoid area and 17 (14.2%) patients had tumour at the post-pharyngeal wall. There were 5 (4.2%) cases with stage I or II disease, 15 (12.5%) cases with stage III disease, 81 (67.5%) cases with stage IVA disease and 19 (15.8%) cases with stage IVB disease. Fourteen (11.7%) patients had synchronous cancer and 22 (18.3%) patients subsequently developed metachronous cancer (Table 1).

**Table 1:** Baseline characteristics of patients and treatment.

Characteristics	Number (%)
**Age (years)**	
**<**55	58 (48.3)
**≥**55	62 (51.7)
**Sex**	
Male	117 (97.5)
Female	3 (2.5)
**Performance status**	
0–1	105 (87.5)
≥2	15 (12.5)
**BMI**	
<18.5	16 (13.3)
≥18.5	104 (86.7)
**Comorbidity score**	
0–1	85 (70.8)
≥2	35 (29.2)
**Smoking (pack-years)**	
<15	19 (22.1)
≥15	67 (77.9)
**Tumour location**	
Pyriform sinus	97 (80.8)
Post-cricoid area	6 (5.0)
Posterior pharyngeal wall	17 (14.2)
**Tumour differentiation**	
Grade 1–2	73 (60.8)
Grade 3	18 (15.0)
Unknown	29 (24.2)
**Stage**	
I-II	5 (4.2)
III	15 (12.5)
IVA	81 (67.5)
IVB	19 (15.8)
**Baseline NLR**	
<4	85 (70.8)
≥4	35 (29.2)
**Treatment modality**	
CCRT	67 (55.8)
IC then CCRT or RT	53 (44.2)
**RT treatment time (days)**	
**<**63	106 (88.3)
≥63	14 (11.7)
**RT response**	
CR	63 (52.5)
Less than CR	57 (47.5)

*BMI, body mass index; Hb, haemoglobin; neutrophil, absolute neutrophil count; lymphocyte, absolute lymphocyte count; NLR, neutrophil-to-lymphocyte ratio; CCRT, concurrent chemoradiotherapy; IC, induction chemotherapy; RT, radiotherapy; CR, complete response.

### Treatment and outcomes

Of the 120 total patients, 117 completed definitive radiotherapy and 3 developed major complications and terminated treatment early. Fifty-three patients received induction chemotherapy followed by radiotherapy or concurrent chemoradiotherapy, while 67 patients underwent concurrent chemoradiotherapy. As multiple prospective studies have not shown a survival difference between concurrent and induction chemotherapy^18^, we analysed all 120 patients together. The median total dose of the cohort was 7020 cGy (range, 700 to 7920 cGy), with a median overall radiotherapy treatment time of 54 days (range, 10 to 94 days). Of the 120 patients, 63 (52.5%) achieved complete response after definitive radiotherapy. The median follow-up time was 24.1 months (range, 3.1 to 111.3 months). The median OS was 31.3 months and the median PFS was 22.5 months.

### Correlation between pretreatment NLR and clinicopathologic characteristics

Before treatment, 35 (29.2%) patients had high NLR (NLR ≥ 4) and 85 (70.8%) patients had low NLR (NLR < 4). High pretreatment NLR (NLR ≥ 4) was significantly correlated with moderate-to-severe anaemia (*p* = 0.02*) and, notably, advanced T classification (*p* = 0.01*) and advanced stage (*p* = 0.02*) (Table 2). Pretreatment NLR was not significantly associated with age, sex, performance status, BMI, smoking history, tumour location, or N classification.

**Table 2:** Relationships between clinicopathological factors and baseline NLR.

Characteristics	Baseline NLR		*p* value
	<4 (n = 85)	≥4 (n = 35)	
**Age (years)**			
<55	40	18	0.81
≥55	45	17	
**Sex**			
Female	3	0	0.56
Male	82	35	
**Performance status**			
0–1	77	28	0.20
≥2	8	7	
**BMI**			
<18.5	9	7	0.28
≥18.5	76	28	
**Comorbidity score**			
0–1	63	22	0.31
≥2	22	13	
**Smoking (pack-years)**			
<15	13	6	0.99
≥15	48	19	
**Baseline Hb (g/dL)**			
<11	4	7	0.02*
≥11	81	28	
**Tumour location**			
Pyriform sinus	69	28	0.97
Post-cricoid area	4	2	
Posterior pharyngeal wall	12	5	
**Tumour differentiation**			
Grade 1-2	47	26	0.42
Grade 3	14	4	
**T classification**			
T1-2	34	5	0.01*
T3-4	51	30	
**N classification**			
N0	9	3	1.00
N1-3	76	32	
**Stage**			
I-II	4	1	0.02*
III	13	2	
IVA	59	22	
IVB	9	10	

*NLR, neutrophil-to-lymphocyte ratio; BMI, body mass index; Hb, haemoglobin; CR, complete response.

### Prognostic significance of baseline NLR and other parameters

**Figure 2:**
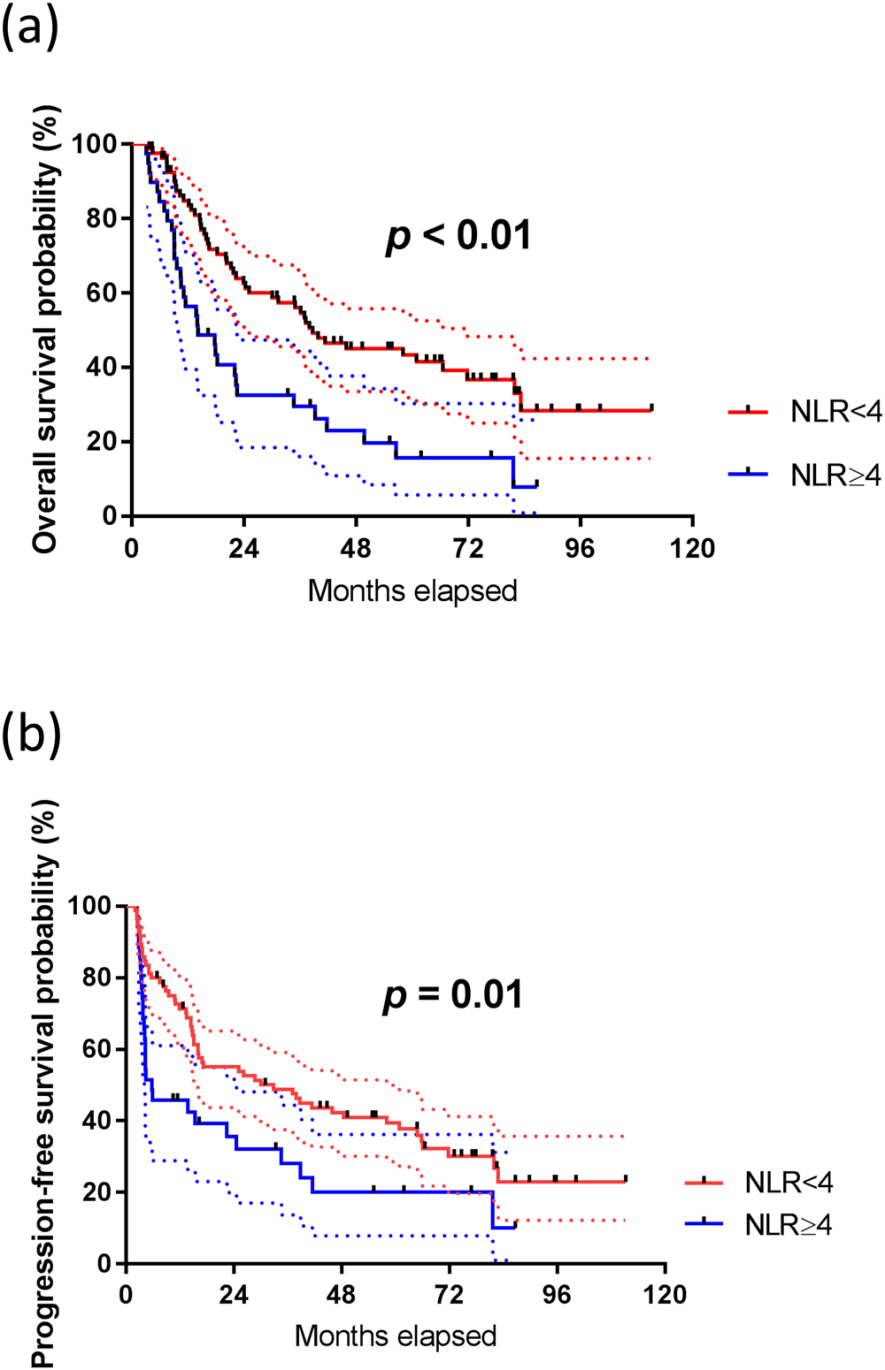
Patients with high NLR had worse (**a**) overall survival and (**b**) progression-free survival in univariate analysis. Dashed lines, 95% confidence interval; p value as calculated by log-rank test.

Analysis of the odds ratio of NLR on treatment response showed that high pretreatment NLR (NLR ≥ 4) was correlated with poor response to definitive chemoradiotherapy (HR = 2.42, 95% CI: 1.08 to 5.44, *p* = 0.03*). In the survival analysis, anaemia was associated with poor overall survival (OS); other known prognostic factors, such as performance status, T classification and overall stage, were associated with both progression-free survival (PFS) and OS. In the univariate analysis, prolonged radiotherapy time and radiotherapy response predicted inferior PFS and OS. NLR was also significantly associated with PFS (Table 3, Fig. 2) and OS (Table 4, Fig. 2). All significant factors in the univariate analysis were then incorporated into the multivariate analysis, except for T classification as it was not independent from overall stage. In the multivariate analysis, pretreatment NLR and treatment response were shown to be independent prognostic factors of PFS (NLR ≥ 4 vs. NLR < 4, HR = 1.71, 95% CI: 1.01 to 2.90, *p* = 0.046*) and overall survival (NLR ≥ 4 vs. NLR < 4, HR = 1.99, 95% CI: 1.21 to 3.28, *p* = 0.01*).

**Table 3:** Univariate and multivariate analysis of prognostic factors associated with progression-free survival (PFS).

Prognostic factor	Univariate analysis			Multivariate analysis		
	HR	95% CI	*p* value	HR	95% CI	*p* value
**Age (years)**						
≥55 vs <55	1.13	0.73–1.73	0.59	—	—	—
**Sex**						
Male vs Female	0.74	0.19–2.79	0.60	—	—	—
**Performance status**						
≥2 vs 0–1	3.19	1.29–7.89	<0.01*	1.50	0.75–2.98	0.25
**BMI**						
≥18.5 vs <18.5	0.72	0.36–1.44	0.30	—	—	**—**
**Comorbidity score**						
≥2 vs 0–1	1.30	0.80–2.11	0.26	—	—	—
**Baseline NLR**						
≥4 vs<4	1.80	1.06–3.04	0.01*	1.71	1.01–2.90	0.046*
**Baseline Hb (g/dL)**						
≥11 vs <11	0.64	0.29–1.41	0.18	—	—	—
**Smoking (pack-years)**						
≥15 vs <15	0.96	0.51–1.83	0.91	—	—	—
**Tumour location**						
Pyriform sinus	1	—	0.81	—	—	—
Post-cricoid area	1.35	0.48–3.80		—	—	—
Post. pharyngeal wall	1.03	0.54–1.94		—	—	—
**T classification**						
T3-4 vs T1-2	1.64	1.06–2.54	0.04*	—	—	—
**N classification**						
N1–3 vs N0	1.55	0.81–2.97	0.26	—	—	—
**Overall stage**						
I-II	1	—	0.01*	0.66	0.16–2.77	0.57
III	1.93	0.72–5.18		0.69	0.32–1.49	0.35
IVA	2.84	1.20–6.76		1	—	—
IVB	5.51	1.86–16.35		1.10	1.57–2.13	0.78
**Tumour differentiation**						
Grade 3 vs Grade 1–2	1.10	0.59–2.05	0.77	—	—	—
**Treatment modality**						
CCRT	1	—	0.60	—	—	—
IC then CCRT or RT	1.12	0.73–1.73		—	—	—
**RT treatment time (days)**						
≥63 vs <63	2.51	1.04–6.06	<0.01*	0.93	0.46–1.89	0.85
**RT response**						
Less than CR vs CR	4.61	2.85–7.46	<0.01*	4.88	2.90–8.21	<0.01*

*BMI, body mass index; NLR, neutrophil-to-lymphocyte ratio; Hb, haemoglobin; IC, induction chemotherapy; CCRT, concurrent chemoradiotherapy; RT, radiotherapy; CR, complete response.

**Table 4:** Univariate and multivariate analysis of prognostic factors associated with overall survival (OS).

Prognostic factor	Univariate analysis			Multivariate analysis		
	HR	95% CI	*p* value	HR	95% CI	*p* value
**Age (years)**						
≥55 vs <55	1.15	0.74–1.78	0.53	—	—	—
**Sex**						
Male vs Female	0.66	0.16–2.68	0.47	—	—	—
**Performance status**						
≥2 vs 0–1	3.62	1.44–9.12	<0.01*	1.79	0.89–3.60	0.10
**BMI**						
≥18.5 vs <18.5	0.64	0.32–1.29	0.14	—	—	—
**Comorbidity score**						
≥2 vs 0–1	1.20	0.74–1.97	0.44	—	—	—
**Baseline NLR**						
≥4 vs <4	2.10	1.23–3.58	<0.01*	1.99	1.21–3.28	0.01*
**Baseline Hb (g/dL)**						
≥11 vs <11	0.46	0.18–1.14	0.02*	1.33	0.64–2.74	0.44
**Smoking (pack-years)**						
≥15 vs <15	0.96	0.49–1.88	0.91	—	—	—
**Tumour location**						
Pyriform sinus	1	—	0.76	—	—	—
Post-cricoid area	1.35	0.48–3.78		—	—	—
Post. pharyngeal wall	1.14	0.59–2.23		—	—	—
**T classification**						
T3-4 vs T1-2	1.91	1.22–3.00	0.01*	—	—	—
**N classification**						
N1-3 vs N0	1.25	0.64–2.44	0.55	—	—	—
**Overall stage**						
I-II	1	—	0.01*	0.78	0.18–3.28	0.73
III	1.99	0.72–5.52		0.79	0.35–1.80	0.58
IVA	2.60	1.07–6.31		1	—	—
IVB	5.52	1.80–16.91		0.97	0.49–1.92	0.94
**Tumour differentiation**						
Grade 3 vs Grade 1–2	1.24	0.63–2.43	0.51	—	—	—
**Treatment modality**						
CCRT	1	—	0.73	—	—	—
IC then CCRT or RT	1.08	0.69–1.68		—	—	—
**RT treatment time (days)**						
≥63 vs <63	2.83	1.16–6.92	<0.01*	0.93	0.46–1.89	0.84
**RT response**						
Less than CR vs CR	5.00	3.07–8.14	<0.01*	5.35	3.08–9.29	<0.01*

*BMI, body mass index; NLR, neutrophil-to-lymphocyte ratio; Hb, haemoglobin; CCRT, concurrent chemoradiotherapy; IC, induction chemotherapy; RT, radiotherapy; CR, complete response.

## Discussion

This study reports three major findings. First, we found that high pretreatment NLR was correlated with advanced T classification and advanced stage. Second, high pretreatment NLR was associated with poor response to definitive chemoradiotherapy. Third, high pretreatment NLR was significantly correlated with worse PFS and OS for HPC patients receiving definitive chemoradiotherapy. To the best of our knowledge, this is the first study of the significance of pretreatment NLR in HPC patients treated with definitive chemoradiotherapy.

NLR can provide insight into the immune-related host response of the tumour microenvironment in various cancers. Elevated NLR levels may result from increased neutrophils and/or decreased lymphocytes. Neutrophils are known to secrete matrix metalloproteinase, vascular endothelial growth factor, fibroblast growth factor, platelet-derived growth factor, TNF-alpha, IL-1 and IL-6^19^. These secreted cytokines and molecules create a microenvironment for extracellular matrix remodelling, endothelial cell migration and tumour cell dissociation that stimulate tumour angiogenesis and contribute to tumour progression^20^. Neutrophils also inhibit the cytolytic activity of active T lymphocytes and natural killer cells^21^. Collectively, high NLR represents an unfavourable tumour microenvironment, which subsequently contributes to tumour growth. In this study of HPC patients, high NLR was significantly associated with advanced T classification and advanced stage. The elevated neutrophil activity and decreased lymphocytic activity may have contributed to accelerated growth of tumour cells. Thus, this is the first study suggesting that NLR is a biomarker of unfavourable tumour microenvironment in HPC patients.

NLR has been reported as a biomarker for predicting therapeutic response in many cancers, regardless of chemotherapy or target therapy^22–24^. In patients with oral cavity squamous cell carcinoma and oesophageal cancer, therapeutic response to radiotherapy-based treatment was better predicted by adding NLR levels^25,26^. Consistent with such previous work, our study revealed that high pretreatment NLR was correlated with poor response to definitive chemoradiotherapy in HPC patients. The result of the current study provides more confidence to physicians in suggesting alternative treatments for HPC patients with high pre-treatment NLR, since poor response is anticipated.

Elevated NLR is associated with poor treatment response as well as with poor survival outcomes. Chua *et al*. reported that high NLR correlated with poor OS in patients with advanced colorectal cancer^19^. Zhou *et al*. revealed that elevated NLR predicted poor PFS and OS in patients with locally advanced oesophageal squamous cell carcinoma treated with definitive chemoradiotherapy^27^. Lo *et al*. reported that high pretreatment NLR correlated with poor prognosis in advanced HPC patients undergoing major surgery^16^. A recent meta-analysis that included 40559 patients revealed a strong prognostic influence of NLR^28^. In line with these previous findings, we also found that elevated NLR was correlated with poor PFS and OS in HPC patients.

We acknowledge that our study is subject to several limitations. First, due to its retrospective nature, selection and survival biases are unavoidable. Thus, our findings warrant further confirmation in a prospective study. In addition, the study population was treated at a single institution and the population was almost exclusively Taiwanese; extrapolation of these results to other Asian or Caucasian populations is necessary and should be done with care.

In conclusion, our study revealed that high pretreatment NLR was associated with host response to tumour aggressiveness. Further, high pretreatment NLR was correlated with poor therapeutic response and was an independent predictor of inferior PFS and OS in HPC patients treated with definitive chemoradiotherapy.

## Methods

### Study population

We retrospectively reviewed the medical records of patients with hypopharyngeal cancer who underwent definitive chemoradiotherapy at National Cheng Kung University Hospital, Taiwan, between September 2009 and October 2015. The inclusion criteria were patients aged over 18 with biopsy-proven hypopharyngeal cancer who had not undergone prior surgery of the hypopharyngeal primary. Patients with initial distant metastases, a history of prior head and neck radiation, treatment with palliative radiation (<60 Gy), or recurrent tumours were excluded. This retrospective study was approved by the National Cheng Kung University Hospital Institutional Review Board, which also granted a waiver of informed consent. This study met the Human Subjects Research Act as required by Taiwan law, and conforms to the Declaration of Helsinki.

### Data collection and definitions of parameters

Previously reported prognostic factors were retrieved from medical records, including age, sex, performance status, body mass index (BMI), comorbidity, smoking history, TNM stage and tumour differentiation. Performance status was documented according to the ECOG system. BMI less than 18.5 was regarded as underweight^29,30^. Comorbidity was scored using the ACE-27 index^31^. The ACE-27 index groups comorbidity conditions as follows: cardiovascular, gastrointestinal, respiratory, renal, neurological, psychiatric, endocrine, immunological, rheumatologic, malignancy prior to index malignancy or concomitant malignancy with index malignancy, substance abuse and obesity. Overall cogent comorbidity was classified by the severity of organ decompensation using four categories: none, mild, moderate, or severe. Smoking history was recorded as packs per year; more than 15 packs/year was regarded as a heavy smoker^32^. Primary tumour classification (T), nodal classification (N) and overall stage were re-defined based on the 7^th^ edition of the American Joint Committee on Cancer (AJCC) staging manual^33^. We also recorded pretreatment blood count data collected within two weeks prior to any treatments. Pretreatment haemoglobin, white blood cell count, absolute neutrophil count and lymphocyte count were extracted. We calculated the neutrophil-to-lymphocyte ratio (NLR) by dividing the absolute neutrophil count by the absolute lymphocyte count. An NLR value of 4 or greater was considered high^34,35^.

### Definitive radiotherapy

All patients were treated with intensity-modulated radiotherapy using 6 or 10 MV linear accelerators (Clinac iX, Varian Medical Systems, Palo Alto, USA). Briefly, a total radiation dose of 7000–7200 cGy (200 cGy/fraction, 5 days per week) was administered to the gross hypopharyngeal tumour and enlarged neck lymph nodes, while 5000 cGy (200 Gy/fraction, 5 days per week) prophylactic irradiation was given to the bilateral neck lymph node regions. All patients were treated by three senior radiation oncologists with over ten years of experience at the same institution according to institutional guidelines. With the same technique and treatment plan of definitive radiotherapy, there were three treatment combination: induction chemotherapy followed by concurrent chemoradiotherapy, concurrent chemoradiotherapy and induction chemotherapy followed by radiotherapy alone. The choice of treatment combination was based on the physician’s judgement.

### Definition of endpoints and statistical considerations

The study endpoints were treatment response, progression-free survival (PFS) and overall survival (OS). Treatment response evaluation was performed six weeks after completion of radiotherapy utilizing the head and neck CT scan. If complete radiological response was achieved, it was recorded as complete response; if it was hard to determine complete radiological response, biopsy was performed for confirmation. If the biopsy was negative for malignancy, response was then documented as complete response (CR); if not, response was documented as less than CR. OS was defined from the date of diagnosis until death from any cause, or censored at last follow-up. PFS was defined from the date of diagnosis to the date of any recurrence or death from any cause, or censored at last follow-up. All variables were grouped as categorical data according to clinically meaningful cut-off values. The Chi-square tests (or Fisher’s exact probability when appropriate) were used for analysing the relationship between NLR and clinicopathological parameters. Odds ratio estimation was used to calculate the relationship between pretreatment NLR and treatment response. For univariate analysis, we plotted survival curves with the Kaplan–Meier method and compared differences between groups using the log-rank test. We evaluated potential prognostic factors with univariate and multivariate regression analysis with respect to PFS and OS; factors with *p* < 0.05 in univariate analysis were selected for inclusion in multivariate analysis. Multivariate analysis was performed using the Cox proportional hazards model. A two-tailed *p* value < 0.05 was considered statistically significant. Statistical analysis was conducted with MedCalc Statistical Software version 15.2.2 (MedCalc Software bvba, Ostend, Belgium). All data generated or analysed during this study are provided with this published article.

## References

[1] Sakaguchi M, Maebayashi T, Aizawa T, Ishibashi N, Saito T (2017). Clinical Outcomes of Hypopharyngeal Cancer Receiving Definitive Radiotherapy with Concurrent Chemotherapy. Anticancer Res.

[2] Nakahara R (2012). Treatment outcomes of definitive chemoradiotherapy for patients with hypopharyngeal cancer. J Radiat Res.

[3] Takehana K (2016). Retrospective analysis of the clinical efficacy of definitive chemoradiotherapy for patients with hypopharyngeal cancer. Jpn J Clin Oncol.

[4] Chang MF (2010). Treatment results for hypopharyngeal cancer by different treatment strategies and its secondary primary–an experience in Taiwan. Radiation oncology (London, England).

[5] Lefebvre JL (2012). Laryngeal preservation with induction chemotherapy for hypopharyngeal squamous cell carcinoma: 10-year results of EORTC trial 24891. Annals of oncology: official journal of the European Society for Medical Oncology.

[6] Wolf GT (1991). Induction chemotherapy plus radiation compared with surgery plus radiation in patients with advanced laryngeal cancer. The New England journal of medicine.

[7] Lefebvre JL (1996). Larynx preservation in pyriform sinus cancer: preliminary results of a European Organization for Research and Treatment of Cancer phase III trial. EORTC Head and Neck Cancer Cooperative Group. Journal of the National Cancer Institute.

[8] Meads MB, Gatenby RA, Dalton WS (2009). Environment-mediated drug resistance: a major contributor to minimal residual disease. Nature reviews. Cancer.

[9] Hanahan D, Weinberg RA (2011). Hallmarks of cancer: the next generation. Cell.

[10] McMillan DC (2013). The systemic inflammation-based Glasgow Prognostic Score: a decade of experience in patients with cancer. Cancer Treat Rev.

[11] Diakos CI, Charles KA, McMillan DC, Clarke SJ (2014). Cancer-related inflammation and treatment effectiveness. The Lancet. Oncology.

[12] Deng Q (2015). Prognostic value of pre-operative inflammatory response biomarkers in gastric cancer patients and the construction of a predictive model. J Transl Med.

[13] Del Prete M (2015). Prognostic clinical factors in pretreated colorectal cancer patients receiving regorafenib: implications for clinical management. Oncotarget.

[14] Keizman D (2012). Pretreatment neutrophil-to-lymphocyte ratio in metastatic castration-resistant prostate cancer patients treated with ketoconazole: association with outcome and predictive nomogram. Oncologist.

[15] Cho IR (2014). Pre-treatment neutrophil to lymphocyte ratio as a prognostic marker to predict chemotherapeutic response and survival outcomes in metastatic advanced gastric cancer. Gastric Cancer.

[16] Lo WC (2017). The Pretreatment Neutrophil-to-Lymphocyte Ratio is a Prognostic Determinant of T3-4 Hypopharyngeal Squamous Cell Carcinoma. Annals of surgical oncology.

[17] Song Y (2015). Preoperative neutrophil-to-lymphocyte ratio as prognostic predictor for hypopharyngeal squamous cell carcinoma after radical resections. The Journal of craniofacial surgery.

[18] Prades JM (2010). Randomized phase III trial comparing induction chemotherapy followed by radiotherapy to concomitant chemoradiotherapy for laryngeal preservation in T3M0 pyriform sinus carcinoma. Acta oto-laryngologica.

[19] Di Carlo E, Forni G, Musiani P (2003). Neutrophils in the antitumoral immune response. Chemical immunology and allergy.

[20] Dumitru CA, Lang S, Brandau S (2013). Modulation of neutrophil granulocytes in the tumor microenvironment: mechanisms and consequences for tumor progression. Seminars in cancer biology.

[21] Shau HY, Kim A (1988). Suppression of lymphokine-activated killer induction by neutrophils. Journal of immunology (Baltimore, Md.: 1950).

[22] Chua W, Charles KA, Baracos VE, Clarke SJ (2011). Neutrophil/lymphocyte ratio predicts chemotherapy outcomes in patients with advanced colorectal cancer. British journal of cancer.

[23] Koti M (2015). A distinct pre-existing inflammatory tumour microenvironment is associated with chemotherapy resistance in high-grade serous epithelial ovarian cancer. British journal of cancer.

[24] Santoni M (2013). Pre-treatment neutrophil-to-lymphocyte ratio may be associated with the outcome in patients treated with everolimus for metastatic renal cell carcinoma. British journal of cancer.

[25] Nakashima H (2016). Pre-treatment neutrophil to lymphocyte ratio predicts the chemoradiotherapy outcome and survival in patients with oral squamous cell carcinoma: a retrospective study. BMC cancer.

[26] Hyder J, Boggs DH, Hanna A, Suntharalingam M, Chuong MD (2016). Changes in neutrophil-to-lymphocyte and platelet-to-lymphocyte ratios during chemoradiation predict for survival and pathologic complete response in trimodality esophageal cancer patients. Journal of Gastrointestinal Oncology.

[27] Zhou XL (2017). Neutrophil-to-lymphocyte ratio as a prognostic biomarker for patients with locally advanced esophageal squamous cell carcinoma treated with definitive chemoradiotherapy. Scientific reports.

[28] Templeton AJ (2014). Prognostic role of neutrophil-to-lymphocyte ratio in solid tumors: a systematic review and meta-analysis. Journal of the National Cancer Institute.

[29] Moon H (2016). Prognostic value of nutritional and hematologic markers in head and neck squamous cell carcinoma treated by chemoradiotherapy. Radiother Oncol.

[30] Fearon K (2011). Definition and classification of cancer cachexia: an international consensus. The Lancet. Oncology.

[31] Sanabria A (2007). Comorbidity is a prognostic factor in elderly patients with head and neck cancer. Annals of surgical oncology.

[32] Lubin JH (2009). Total exposure and exposure rate effects for alcohol and smoking and risk of head and neck cancer: a pooled analysis of case-control studies. Am J Epidemiol.

[33] Edge SB, Compton CC (2010). The American Joint Committee on Cancer: the7th edition of the AJCC cancer staging manual and the future of TNM. Annals of surgical oncology.

[34] Halazun KJ (2008). Elevated preoperative neutrophil to lymphocyte ratio predicts survival following hepatic resection for colorectal liver metastases. Eur J Surg Oncol.

[35] Kishi Y (2009). Blood neutrophil-to-lymphocyte ratio predicts survival in patients with colorectal liver metastases treated with systemic chemotherapy. Annals of surgical oncology.

